# Attenuation of Circulating Trimethylamine N-Oxide Prevents the Progression of Cardiac and Renal Dysfunction in a Rat Model of Chronic Cardiorenal Syndrome

**DOI:** 10.3389/fphar.2021.751380

**Published:** 2021-10-14

**Authors:** Deling Zou, Yanyu Li, Guangping Sun

**Affiliations:** ^1^ Department of Cardiology, Shengjing Hospital, China Medical University, Shenyang, China; ^2^ Department of Nephrology, Binzhou People’s Hospital, Binzhou, China; ^3^ Department of Nephrology, Shengjing Hospital, China Medical University, Shenyang, China

**Keywords:** chronic heart failure, chronic kidney disease, cardiorenal syndrome-2, trimethylamine N-oxide, inflammation

## Abstract

Chronic heart failure (HF) frequently causes progressive decline in kidney function, known as cardiorenal syndrome-2 (CRS2). Current treatment options for CRS2 remain unacceptably limited. Trimethylamine-N-oxide (TMAO), a metabolite of gut microbiota, has recently been implicated in the pathogenesis of both HF and chronic kidney disease. Here we examined whether circulating TMAO is elevated in CRS2 and if so, whether attenuation of circulating TMAO would ameliorate the progression of CRS2. Sprague-Dawley rats underwent surgery for myocardial infarction (MI) or sham (week 0) followed by subtotal (5/6) nephrectomy (STNx) or sham at week 4 to induce CRS2 or control. At week 6, MI + STNx rats and control rats received vehicle or 1.0% 3,3-Dimethyl-1-butanol (DMB, a TMAO inhibitor) treatment for 8 weeks. Compared with control rats, MI + STNx rats exhibited elevated serum TMAO at week 6, which was increased further at week 14 but was attenuated by DMB treatment. MI + STNx rats showed cardiac dysfunction as assessed by echocardiography and renal dysfunction as evidenced by increased serum creatinine and urinary kidney injury molecule-1 and decreased creatinine clearance at week 6. The cardiac and renal dysfunction in MI + STNx rats was exacerbated at week 14 but was prevented by DMB treatment. Molecular and histological studies revealed myocyte hypertrophy and increases in interstitial myocardial fibrosis and gene expression of pro-hypertrophic and pro-fibrotic markers in both heart and kidney at week 14, which were accompanied by elevated gene expression of proinflammatory cytokines. The changes in molecular and histological parameters observed in MI + STNx rats were significantly reduced by DMB treatment. These findings suggest that rats with CRS2 have elevated circulating TMAO, which is associated with the exacerbation of cardiac and renal dysfunction. Attenuation of circulating TMAO can ameliorate cardiac and renal injury and prevents the progression of CRS2.

## Introduction

Heart failure (HF) is a growing health problem and a major cause of mortality and morbidity in the world. Patients with HF frequently develop secondary kidney impairment, resulting in a vicious cycle that exaggerates the decline in both cardiac and renal function. Kidney impairment arising from a primary defect in the heart has been defined as cardiorenal syndrome type 2 (CRS2) (Ronco et al., 2008; Savira et al., 2020a). The prevalence of renal impairment in patients with HF is approximately 26% and the degree of renal dysfunction is a powerful independent risk factor for all-cause mortality in patients with HF (Uduman, 2018). Clinical studies have shown that even a slight worsening of renal function in patients with HF is associated with increased mortality and prolonged hospitalization (Gottlieb et al., 2002). Due to the dual impairment of the heart and the kidney in CRS2, definitive therapeutic options remain suboptimal and often empirical as no evidence-based treatment guidelines currently exist (Ronco et al., 2010; Muhlberger et al., 2012). Thus, understanding the precise mechanisms and pharmacologically targeting a common pathway involved in both cardiac and renal pathophysiology may be a promising strategy in the CRS2 setting.

Accumulating evidence reveals that alterations in gut microbiota are associated with many disease states (Albert and Tang, 2018; Subramaniam and Fletcher, 2018; Feng et al., 2019; Li et al., 2021). Trimethylamine N-oxide (TMAO), a molecule generated from dietary choline, betaine, and carnitine via gut microbial metabolism, has recently emerged as an important contributor to the pathogenesis of cardiovascular and renal diseases, including HF and chronic kidney disease (CKD) (Tang et al., 2015; Kim et al., 2016; Kitai et al., 2016; Subramaniam and Fletcher, 2018; Anselmi et al., 2020; Mamic et al., 2020; Steinke et al., 2020). For example, circulating TMAO levels are elevated in patients with HF and associated with disease severity, ischemic aetiology and poor survival (Troseid et al., 2015). Administration of TMAO exacerbates pressure overload-induced HF in mice (Organ et al., 2016). TMAO could enhance patient susceptibility to HF by increasing myocardial fibrosis (Schuett et al., 2017). In addition, TMAO has been considered a strong biomarker for CKD risk and a contributor to the pathogenesis of CKD (Tang et al., 2015; Kim et al., 2016; Mafune et al., 2016; Missailidis et al., 2016; Brown and Hazen, 2017). Patients with CKD have elevated circulating TMAO levels, which are associated with a 2.8-fold increased mortality risk (Tang et al., 2015). Mice fed with choline (a TMAO precursor) or TMAO show renal tubulointerstitial fibrosis and dysfunction (Mafune et al., 2016). We previously reported that elevated TMAO contributed to renal impairment in a mouse model of diet-induced obesity (Sun et al., 2017). A new clinical study shows that elevated circulating TMAO is associated with renal dysfunction in patients with HF with preserved ejection fraction (Guo et al., 2020). In the present study, we examined whether circulating TMAO levels are elevated in CRS2 and if so, whether attenuation of circulating TMAO levels would ameliorate the progression of cardiac and renal dysfunction in CRS2. For this purpose, we used a rat model of chronic CRS2, which mimics human phenotypes.

## Methods

### Animals

Adult male Sprague-Dawley rats weighing 250–300 g were purchased from Beijing Laboratory Animal Research Center (Beijing, China) and were housed in a temperature-controlled (23°C–25°C) room under a 12-h light/dark cycle. Standard rat chow and water were provided ad libitum. Animal procedures and protocols were performed in accordance with the “Guiding Principles for Research Involving Animals and Human Beings”, and they were approved by the Institutional Animal Care and Use Committee at China Medical University.

### Protocol

The animal model of CRS2 was surgically generated by combined coronary artery ligation-induced myocardial infarction (MI) and subtotal (5/6) nephrectomy (STNx) (Liu et al., 2013). This animal model has been considered a useful model to assess pathophysiology and mechanisms underlying CRS2 as well as the effects of potential therapies in this setting. In this model, STNx promotes cardiac remodeling (hypertrophy and fibrosis) and renal fibrosis, while MI facilitates STNx-induced renal fibrosis (Liu et al., 2013). The characteristics of this model recapitulate many clinical features of CRS2 patients (Liu et al., 2013). Briefly, rats underwent coronary artery ligation to induce MI (*n* = 30) or sham operation (SHAM, *n* = 16). Four weeks after MI or SHAM surgery, MI rats received STNx surgery while SHAM-operated MI rats received a SHAM-operated STNx procedure. Two weeks later, MI + STNx rats and SHAM + SHAM rats were treated with either vehicle (VEH, tap water) or 1.0% 3,3-Dimethyl-1-butanol (DMB, an inhibitor of trimethylamine formation) in drinking water for 8 weeks. Eleven MI + STNx rats died before the end of the study protocol and these animals were excluded from the study. Thus, final study groups from which data were acquired were: MI + STNx + VEH (*n* = 10), MI + STNx + DMB (*n* = 9), SHAM + SHAM + VEH (*n* = 8), and SHAM + SHAM + DMB (*n* = 8). The dose of DMB used in the present study has been demonstrated to effectively inhibit TMA formation and attenuate circulating TMAO levels in rodents (Sun et al., 2017; Li X. et al., 2019). Before DMB treatment (week 6 after MI) and at the termination of the study protocol (week 14 after MI), tail cuff plethysmography was used to measure systolic blood pressure and echocardiography was performed to assess cardiac function. Blood or 24 h urine were collected to assess serum TMAO levels or renal function. After euthanasia, heart, lung and kidney tissues were collected for anatomic analysis or molecular studies.

### Induction of Myocardial Infarction

MI was induced by left coronary artery ligation as previously described (Li X. et al., 2019; Savira et al., 2020b). Briefly, rats were intubated and ventilated with a rodent ventilator under anesthesia with ketamine/xylazine (100:10 mg/kg; ip). An anterior thoracotomy was performed, and the heart was exposed through the fourth intercostal space. A 6–0 silk suture was placed through the myocardium into the anterolateral left ventricular wall around the left anterior descending coronary artery. The silk suture was then tied resulting in permanent ligation of the left anterior descending coronary artery. SHAM rats underwent the same procedure without ligation of the left anterior descending coronary artery.

### Induction of Chronic Kidney Disease

Four weeks after MI, rats were anesthetized with ketamine/xylazine (100:10 mg/kg; ip). SHAM and MI rats were subjected to either SHAM operation or STNx surgery respectively, as previously described (Savira et al., 2020b). In brief, unilateral right nephrectomy was performed followed by selective ligation of two of 3–4 extra-renal branches of the left renal artery. The SHAM group underwent laparotomy and manipulation of both kidneys before wound closure.

### Echocardiographic Assessment of Cardiac Function

Infarct size and cardiac function after MI were assessed by transthoracic echocardiography as previously described (Li X. et al., 2019). In brief, rats were anesthetized with ketamine (60 mg/kg ip) and the parasternal long-axis and short-axis views of the left ventricular (LV) at the level of the papillary muscle tips were obtained from two-dimensional echocardiograms. Two-dimensionally targeted M-mode tracings were recorded through the anterior and posterior walls. LV internal diameter at end-systole and end-diastole, LV end-systolic area and LV end-diastolic area were measured. LV end-systolic volume (LVESV), LV end-diastolic volume (LVEDV), LV fractional shortening (LVFS), LV ejection fraction (LVEF) and LV ischemic area (LVIA) were calculated.

### Assessment of Renal Function

Rats were individually kept in metabolic cages for 24 h with free access to water and food. Urine samples were collected and the volume was measured. Blood samples were obtained from tail vein. Urinary and serum creatinine concentrations were measured using a rat creatinine assay kit (Crystal Chem Inc., IL, United States). Creatinine clearance was calculated using a standard formula. Albumin in urine was assessed using a rat microalbumin ELISA (Biocompar, CA, United States). Urinary kidney injury molecule-1 (Kim-1) was measured using a rat kim-1 ELISA kit (Abcam, MA, United States).

### Quantification of mRNA Expression

Total RNA was extracted from the non-infarct area of the heart and kidney tissues using RNeasy plus mini kit (QIAGEN China Co. Ltd., Shanghai, China). mRNA expression of pro-hypertrophic markers atrial natriuretic peptide (ANP), brain natriuretic peptide (BNP) and β-myosin heavy chain (β-MHC) in the heart, mRNA expression of pro-fibrotic markers transforming growth factor (TGF)-β1, collagen I, collagen III and tissue inhibitors of metalloproteinases (TIMP)-2 in the heart, mRNA expression of pro-fibrotic markers TGF-β1, collagen I and collagen IV in the kidney, and mRNA expression of proinflammatory cytokines tumor necrosis factor (TNF)-α, interleukin (IL)-6 and IL-8 in the heart and kidney, were analyzed with SYBR Green real-time PCR after reverse transcription of total RNA. Sequences for each primer pair are shown in Table 1. The ABI Prism 7,000 sequence detection system (Applied Biosystems, Carlsbad, CA) was used to perform real-time PCR. mRNA data were normalized by β-actin and expressed as fold changes relative to SHAM + SHAM + VEH group.

**TABLE 1:** Sequences for primers.

*Gene*	*Primers*	*Sequences*
ANP	Forward primer	5′-ATC​TGA​TGG​ATT​TCA​AGA​ACC-3′
	Reverse primer	5′-CTC​TGA​GAC​GGG​TTG​ACT​TC-3′
BNP	Forward primer	5′-ACA​ATC​CAC​GAT​GCA​GAA​GCT-3′
	Reverse primer	5′-GGG​CCT​TGG​TCC​TTT​GAG​A-3′
β-MHC	Forward primer	5′-TTG​GCA​CGG​ACT​GCG​TCA​TC-3′
	Reverse primer	5′-GAG​CCT​CCA​GAG​TTT​GCT​GAA​GGA-3′
TGF-β1	Forward primer	5′-AGA​AGT​CAC​CCG​CGT​GCT​AA-3′
	Reverse primer	5′-TCC​CGA​ATG​TCT​GAC​GTA​TTG-3′
collagen I	Forward primer	5′-CAT​GTT​CAG​CTT​TGT​GGA​CCT-3′
	Reverse primer	5′-GCA​GCT​GAC​TTC​AGG​GGA​TGT-3′
collagen III	Forward primer	5′-GGG​ATC​CAA​TGA​GGG​AGA​AT-3′
	Reverse primer	5′-GCT​CCA​TTC​ACC​AGT​GTG​TTT-3′
collagen IV	Forward primer	5′-GAG​GGT​GCT​GGA​CAA​GCT​CTT-3′
	Reverse primer	5′-TAA​ATG​GAC​TGG​CTC​GGA​ATT​C-3′
TIMP-2	Forward primer	5′-TAT​TGT​GCC​CTG​GGA​CAC​G-3′
	Reverse primer	5′-GTC​CAT​CCA​GAG​GCA​CTC​ATC-3′
TNF-α	Forward primer	5′-CCT​TAT​CTA​CTC​CCA​GGT​TCT​C-3′
	Reverse primer	5′-TTT​CTC​CTG​GTA​TGA​ATG​GC-3′
IL-6	Forward primer	5′-TCC​TAC​CCC​AAC​TTC​CAA​TGC​TC-3′
	Reverse primer	5′-TTG​GAT​GGT​CTT​GGT​CCT​TAG​CC-3′
IL-8	Forward primer	5′-GTG​CAG​TTT​TGC​CAA​GGA​GT-3′
	Reverse primer	5′-TTA​TGA​ATT​CTC​AGC​CCT​CTT​CAA​AAA​CTT CTC-3′
β-actin	Forward primer	5′-CCG​CGA​GTA​CAA​CCT​TCT-3′
	Reverse primer	5′-CGT​CAT​CCA​TGG​CGA​ACT-3′

### Histological Studies

Masson Trichrom staining was performed to evaluate fibrosis and collagen deposits in the heart and kidney tissues using a Masson Trichrom stain kit (Thermo Scientific, Rockford, IL, United States). Quantitation of fibrosis was performed on non-infarct area of the LV and kidney. Fibrosis was calculated based upon percentages of collagen positive areas in the total tissue area as described previously (Sun et al., 2017). Hematoxylin-eosin staining was applied to determine myocyte cross-sectional area in the non-infarct zone of the LV sub-endocardium as described previously (Liu et al., 2013). Fifty myocytes with equal-sized nuclei and intact cellular membranes in each LV were selected. Cell surface areas were calculated by measuring circumferential length of the myocyte and measurements from each rat were averaged for data analysis.

### Measurements of Serum Trimethylamine-N-Oxide Levels

Serum TMAO levels were analyzed using a commercially available ELISA kit (MyBioSource, Inc. San Diego, CA, United States) according to the manufacturers’ instructions.

### Statistical Analysis

All data were presented as mean ± SE. Statistical analyses were performed using GraphPad Software (La Jolla, CA, United States). Differences between groups were analyzed with two-way ANOVA followed by Tukey post hoc tests. *p* < 0.05 was considered statistically significant.

## Results

### Effects of Dimethyl-1-Butanol Treatment on Circulating Trimethylamine-N-Oxide Levels

**FIGURE 1:**
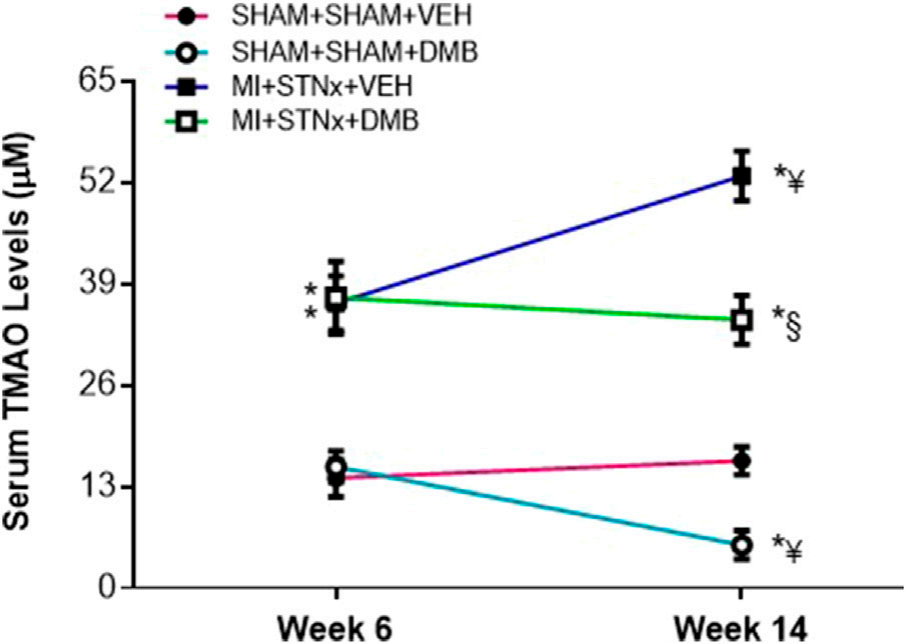
Effects of cardiorenal syndrome type 2 (CRS2) and 3,3-Dimethyl-1-butanol (DMB, an inhibitor of trimethylamine formation) treatment on serum TMAO levels. CRS2 was induced by myocardial infarction (MI) followed by 5/6 subtotal nephrectomy (STNx). Sham MI (SHAM) and sham STNx (SHAM) animals served as control. VEH: vehicle treatment. Data are expressed as mean ± SE (*n* = 8–10 for each group). *p < 0.05 vs SHAM + SHAM + VEH at each time point; ¥ *p* < 0.05 vs week 6; §*p* < 0.05, MI + STNx + DMB vs MI + STNx + VEH.

As shown in Figure 1, serum TMAO levels were markedly higher in the two MI + STNx groups compared with their respective SHAM + SHAM groups at week 6 (before DMB treatment). There were no differences in serum TMAO levels between the two MI + STNx groups or between the two SHAM + SHAM groups at this time point. Serum TMAO levels in VEH-treated SHAM + SHAM rats were not changed, whereas serum TMAO levels in VEH-treated MI + STNx rats increased further at week 14, compared with those at week 6. Notably, DMB treatment for 8 weeks significantly prevented further increases in serum TMAO levels in MI + STNx rats and reduced serum TMAO levels in SHAM + SHAM rats.

Effects of DMB treatment on echocardiographic variables, blood pressure, heart rate and anatomic parameters.

**FIGURE 2:**
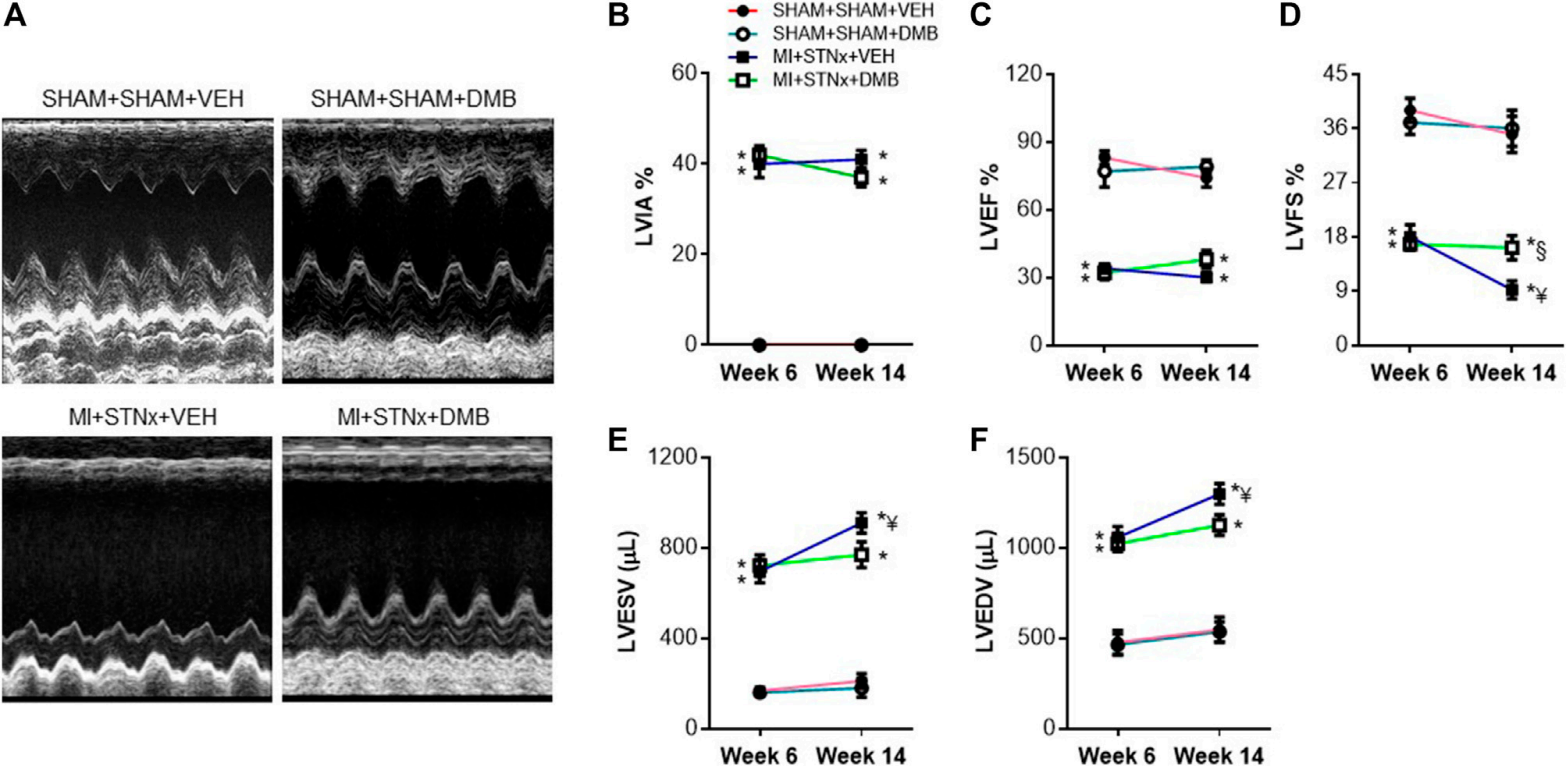
Representative M-mode echocardiograms obtained with two-dimensional guidance from a long-axis ventricular view in each group **(A)** and effects of CRS2 and DMB treatment on echocardiographic variables including percent left ventricular ischemic area (LVIA%) **(B)**, LV ejection fraction (LVEF) **(C)**, LV fractional shortening (LVFS) **(D)**, LV volumes at end cardiac systole (LVESV) **(E)** and LV volumes at end cardiac diastole (LVEDV) **(F)**. Data are expressed as mean ± SE (*n* = 8–10 for each group). *p < 0.05 vs SHAM + SHAM + VEH at each time point; ¥ *p* < 0.05 vs week 6; §*p* < 0.05, MI + STNx + DMB vs MI + STNx + VEH.

Echocardiographic assessment revealed that LVIA% was similar in the two MI + STNx groups at week 6 and was not altered at week 14 (Figure 2B). No significant differences in LVEF (Figure 2C), LVFS (Figure 2D), LVESV (Figure 2E) and LVEDV (Figure 2F) were observed between the two SHAM + SHAM groups throughout the experimental protocol. The LVEF and LVFS were significantly decreased, while LVESV and LVEDV were increased in the two MI + STNx groups at week 6, compared with their respective SHAM + SHAM groups. However, there were no differences in above echocardiographic variables between the two MI + STNx groups at week 6. Of note, LVFS decreased further, whereas LVESV and LVEDV increased further in VEH-treated MI + STNx rats but not in DMB-treated MI + STNx rats at week 14, compared with those at week 6. LVEF tended to be higher in DMB-treated MI + STNx rats than VEH-treated MI + STNx rats at week 14, but the difference did not reach statistical significance.

As presented in Table 2, heart rate and systolic blood pressure were comparable among 4 experimental groups at week 6 and week 14. The BW was markedly lower in both MI + STNx groups than their respective SHAM + SHAM groups, and no difference in BW was found between the two MI + STNx groups or between the two SHAM + SHAM groups at week 14. The LV/tibial length, RV/tibial length and lung/tibial length in VEH-treated MI + STNx rats, compared with VEH-treated SHAM + SHAM rats, were significantly increased, but were reduced in DMB-treated MI + STNx rats at week 14. DMB treatment had no effects on above anatomic parameters in SHAM + SHAM rats.

**TABLE 2:** Heart rate, blood pressure and anatomic variables.

	SHAM + SHAM + VEH (*N* = 8)	SHAM + SHAM + DMB (*n* = 8)	MI + STNx + VEH (*n* = 10)	MI + STNx + DMB (*n* = 9)
Week 6				
***Heart rate (Beats/min)***	372 ± 15	380 ± 20	378 ± 13	391 ± 22
***Blood pressure (mmHg)***	132 ± 11	125 ± 13	147 ± 18	154 ± 15
Week 14				
***Heart rate (Beats/min)***	386 ± 25	377 ± 19	395 ± 23	387 ± 16
***Blood pressure (mmHg)***	138 ± 20	130 ± 19	155 ± 21	148 ± 27
***Body weight (g)***	429 ± 5	435 ± 9	372 ± 7*	377 ± 5*
***LV/tibial length (mg/cm)***	221 ± 4	230 ± 5	296 ± 10*	261 ± 8*§
***RV/tibial length (mg/cm)***	48 ± 2	47 ± 2	93 ± 8*	72 ± 5*§
***Lung/tibial length (mg/cm)***	325 ± 5	316 ± 6	857 ± 76*	514 ± 38*§

LV: left ventricular; RV: right ventricular; MI: myocardial infarction; STNx: 5/6 subtotal nephrectomy; VEH: vehicle; DMB: 1.0% 3,3-Dimethyl-1-butanol (DMB, a TMAO inhibitor).

a *p* < 0.05 vs SHAM + SHAM + VEH or SHAM + SHAM + DMB; §*p* < 0.05 vs MI + STNx.

### Effects of Dimethyl-1-Butanol Treatment on Renal Function

**FIGURE 3:**
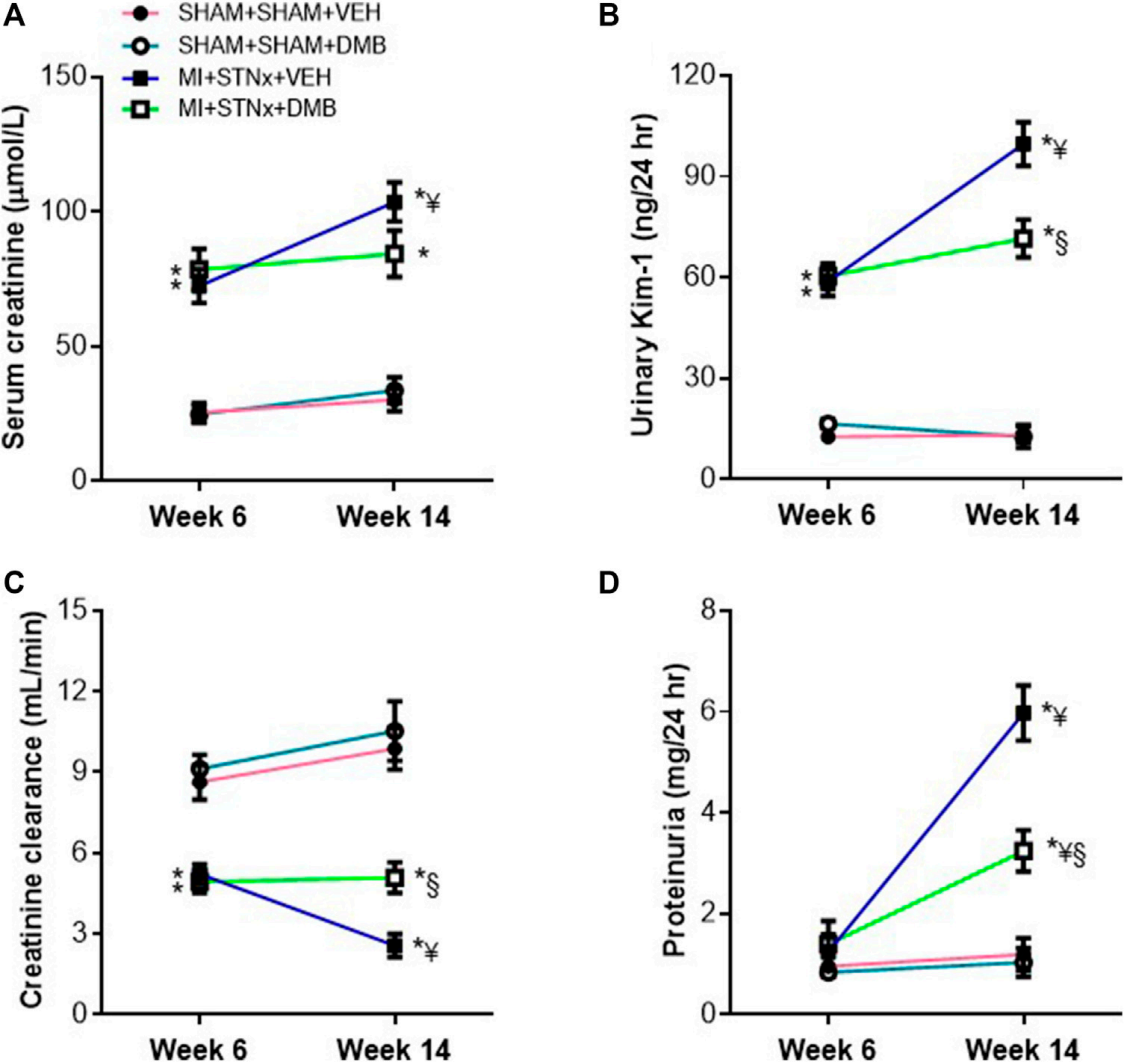
Effects of CRS2 and DMB treatment on renal function assessed by serum creatinine concentrations **(A)**, urinary kidney injury molecule-1 (Kim-1) **(B)**, creatinine clearance **(C)** and proteinuria **(D)**. Data are expressed as mean ± SE (*n* = 8–10 for each group). *p < 0.05 vs SHAM + SHAM + VEH at each time point; ¥ *p* < 0.05 vs week 6; §*p* < 0.05, MI + STNx + DMB vs MI + STNx + VEH.

The levels of serum creatinine (Figure 3A) and urinary KIM-1 (Figure 3B) were significantly higher, but creatinine clearance (Figure 3C) was lower in VEH-treated MI + STNx rats, compared with VEH-treated SHAM + SHAM rats at week 6. The levels of serum creatinine and urinary KIM-1 increased further, whereas creatinine clearance decreased further in VEH-treated MI + STNx rats but not in DMB-treated MI + STNx rats at week 14, compared with those at week 6. There was no significant difference in proteinuria among 4 experimental groups at week 6 (Figure 3D). At week 14, proteinuria was significantly elevated in both MI + STNx groups than their respective SHAM + SHAM groups, but the increase was less in DMB-treated MI + STNx rats than VEH-treated MI + STNx rats.

### Effects of Dimethyl-1-Butanol Treatment on Myocyte Hypertrophy and Cardiac and Renal Fibrosis

**FIGURE 4:**
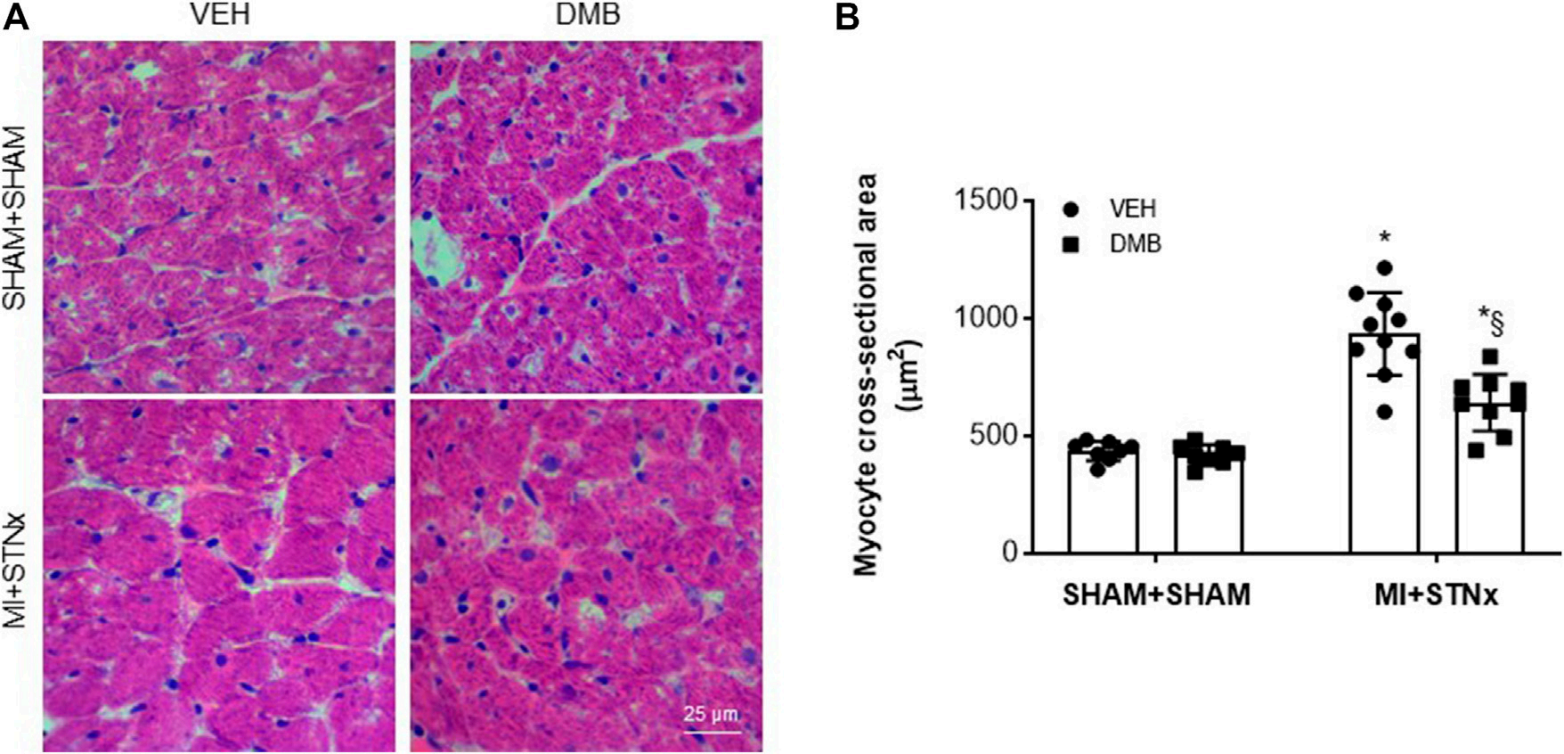
Representative hematoxylin eosin staining of the heart sections. **(A)** and effects of CRS2 and DMB treatment on cardiac myocyte cross-sectional area (a marker of myocyte hypertrophy) in the heart **(B)**. Data are expressed as mean ± SE (*n* = 8–10 for each group). *p < 0.05 vs SHAM + SHAM + VEH; §*p* < 0.05, MI + STNx + DMB vs MI + STNx + VEH.

Compared with VEH-treated SHAM + SHAM rats, cardiac myocyte cross-sectional area in the non-infarct zone (Figure 4) was significantly increased in VEH-treated MI + STNx rats, and this was attenuated by DMB treatment. DMB treatment did not alter cardiac myocyte cross-sectional area in SHAM + SHAM rats.

**FIGURE 5:**
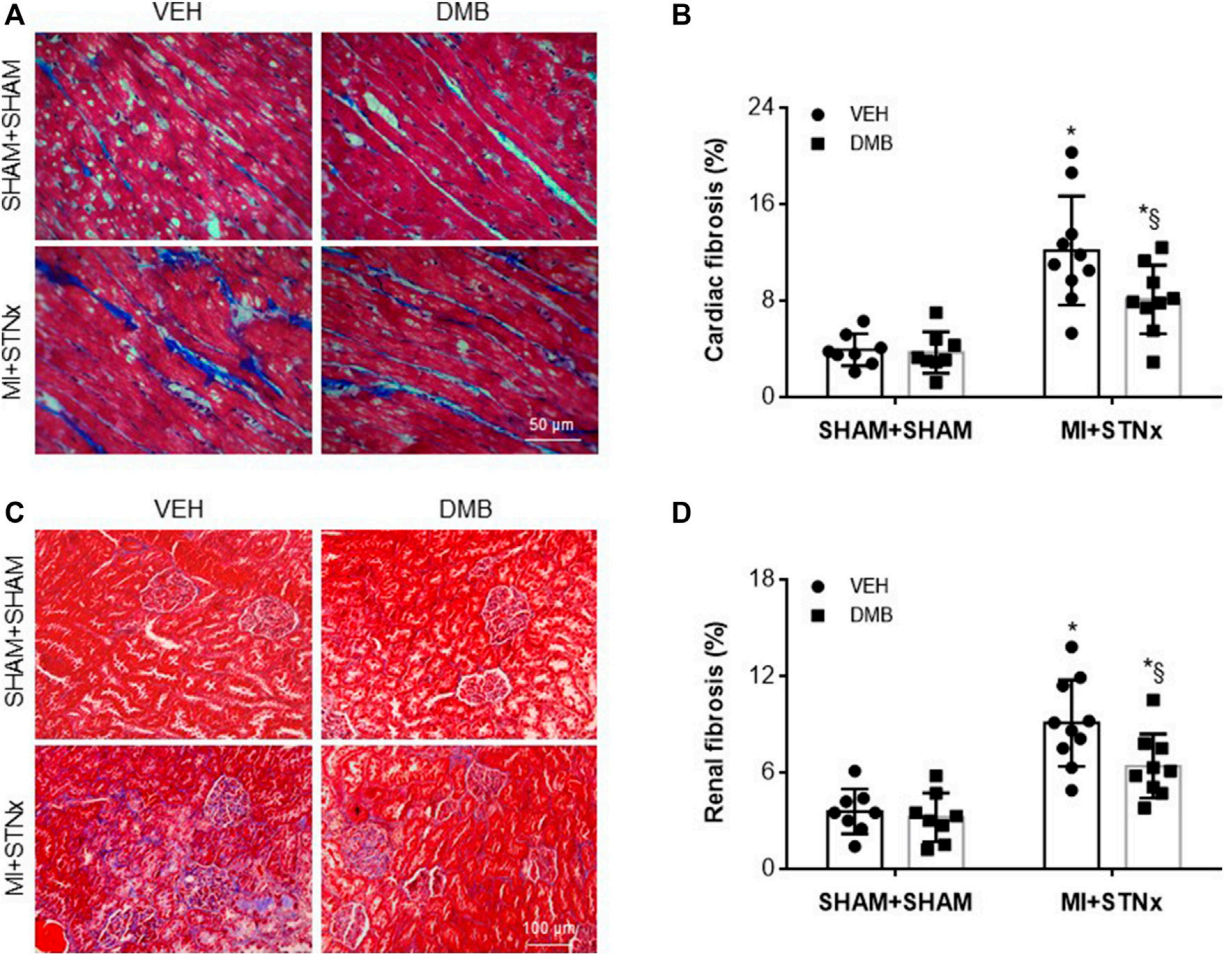
Representative Masson Trichrome staining of the heart **(A)** and kidney **(C)** sections and effects of CRS2 and DMB treatment on cardiac **(B)** and renal fibrosis **(D)**. Data are expressed as mean ± SE (*n* = 8–10 for each group). *p < 0.05 vs SHAM + SHAM + VEH; §*p* < 0.05, MI + STNx + DMB vs MI + STNx + VEH.

Both cardiac fibrosis in the non-infarct zone (Figures 5A,B) and renal fibrosis (Figures 5C,D) were significantly elevated in VEH-treated MI + STNx rats compared with VEH-treated SHAM + SHAM rats. DMB treatment reduced cardiac and renal fibrosis in MI + STNx rats but had no effect in SHAM + SHAM rats.

Effects of DMB treatment on gene expression of pro-hypertrophic and pro-fibrotic markers.

**FIGURE 6:**
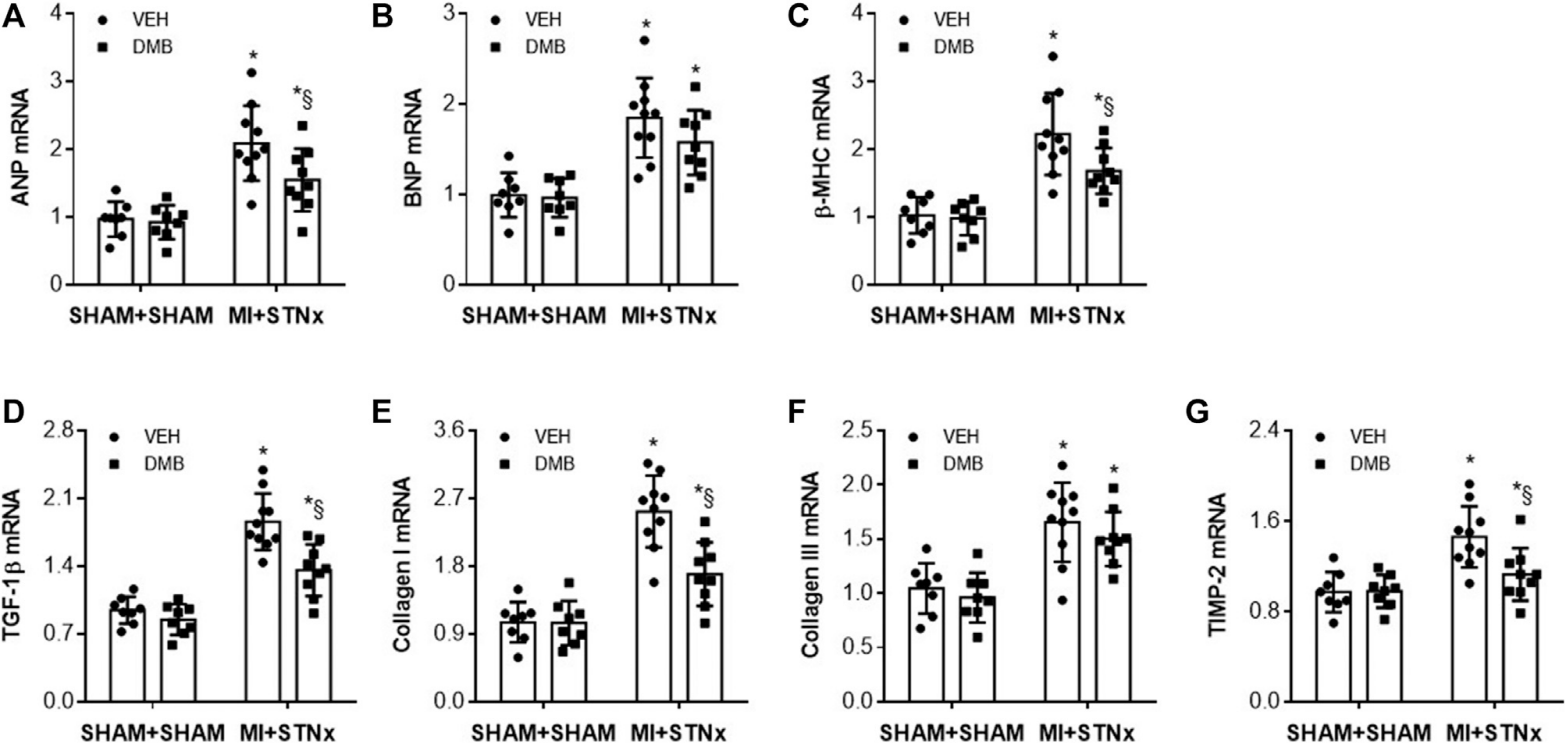
Effects of CRS2 and DMB treatment on gene expression of pro-hypertrophic markers ANP **(A)**, BNP **(B)** and β-MHC **(C)** and on gene expression of pro-fibrotic markers TGF-β1 **(D)**, Collagen-I **(E)**, collagen-III **(F)** and TIMP-2 **(G)** in the heart. Data are expressed as mean ± SE (*n* = 8–10 for each group). *p < 0.05 vs SHAM + SHAM + VEH; §*p* < 0.05, MI + STNx + DMB vs MI + STNx + VEH.

At week 14, we observed significant increases in gene expression of pro-hypertrophic markers ANP (Figure 6A), BNP (Figure 6B) and β-MHC (Figure 6C), and gene expression of pro-fibrotic markers TGF-β (Figure 6D), Collagen-I (Figure 6E), collagen-III (Figure 6F) and TIMP-2 (Figure 6G) in the non-infarct zone in VEH-treated MI + STNx rats compared with VEH-treated SHAM + SHAM rats. DMB treatment of MI + STNx rats decreased gene expression of ANP, β-MHC, TGF-β, Collagen-I and TIMP-2 but did not alter gene expression of BNP and collagen-III. No change in above gene expression was found in SHAM + SHAM rats after DMB treatment.

**FIGURE 7:**
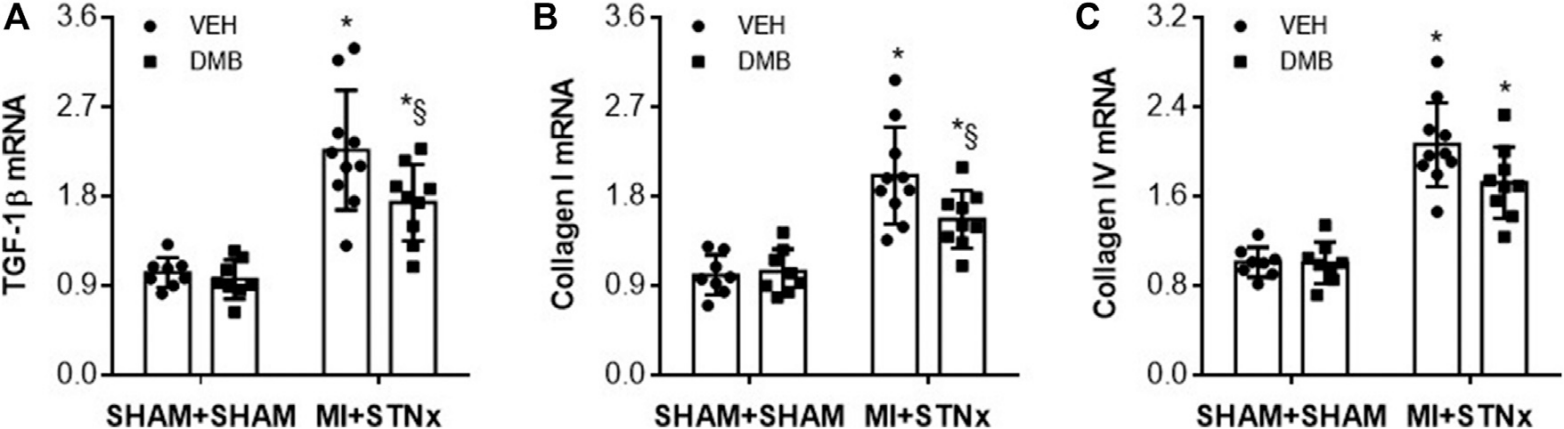
Effects of CRS2 and DMB treatment on gene expression of pro-fibrotic markers TGF-β1 **(A)**, Collagen-I **(B)** and collagen-IV **(C)** in the kidney. Data are expressed as mean ± SE (*n* = 8–10 for each group). *p < 0.05 vs SHAM + SHAM + VEH; §*p* < 0.05, MI + STNx + DMB vs MI + STNx + VEH.

Gene expression of TGF-β (Figure 7A), collagen-I (Figure 7B) and collagen-IV (Figure 7C) in the kidney was also significantly increased in VEH-treated MI + STNx rats as compared to VEH-treated SHAM + SHAM rats. DMB treatment of MI + STNx rats reduced gene expression of TGF-β and collagen-I without effect on collagen-IV. Gene expression of these pro-fibrotic markers was unchanged in SHAM + SHAM rats after DMB treatment.

### Effects of DMB Treatment on Gene Expression of Proinflammatory Cytokines

**FIGURE 8:**
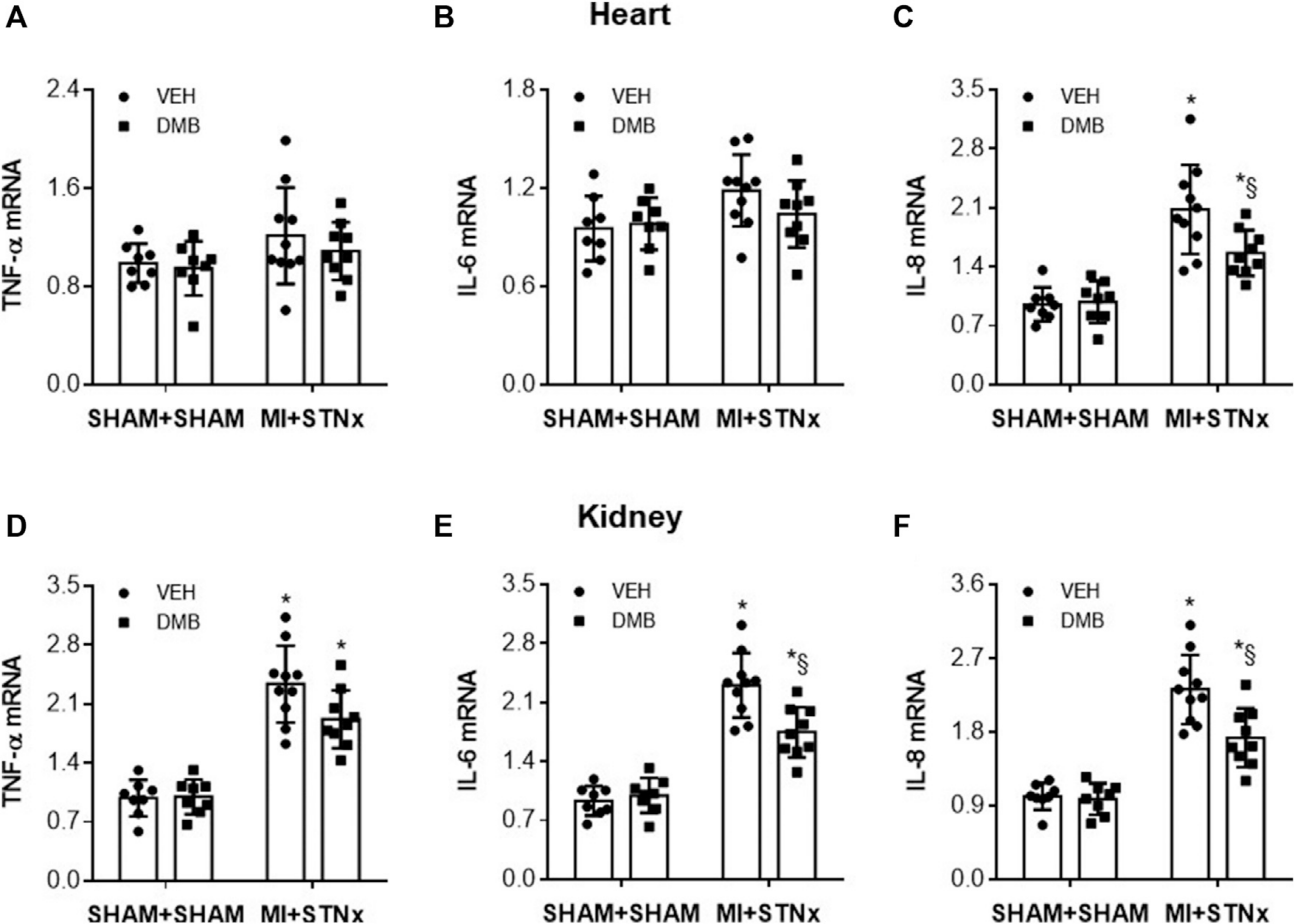
Effects of CRS2 and DMB treatment on gene expression of pro-inflammatory cytokines tumor necrosis factor (TNF)-α **(A)** and **(D)** and interleukin (IL)-6 **(B)** and **(E)** and IL-8 **(C)** and **(F)** in the heart and kidney, respectively. Data are expressed as mean ± SE (*n* = 8–10 for each group). *p < 0.05 vs SHAM + SHAM + VEH; §*p* < 0.05, MI + STNx + DMB vs MI + STNx + VEH.

Gene expression of proinflammatory cytokines TNF-α (Figure 8A) and IL-6 (Figure 8B) in the heart was comparable among the 4 experimental groups. However, Gene expression of IL-8 (Figure 8C) was markedly higher in the non-infarct zone in VEH-treated MI + STNx rats than VEH-treated SHAM + SHAM rats. The increase in gene expression of IL-8 in MI + STNx rats was reduced by DMB treatment, which had no effect in SHAM + SHAM rats.

Gene expression of TNF-α (Figure 8D), IL-6 (Figure 8E) and IL-8 (Figure 8F) was significantly increased in the kidney in VEH-treated MI + STNx rats compared with VEH-treated SHAM + SHAM rats. DMB treatment did not altered gene expression of TNF-α but attenuated gene expression of IL-6 and IL-8 in the kidney of MI + STNx rats. DMB treatment did not changed any of these measured proinflammatory cytokines in SHAM + SHAM rats.

## Discussion

The major findings of this study are: 1) Rats with CRS2 exhibit elevated levels of circulating TMAO, which can be attenuated by treatment with TMAO inhibitor DMB; 2) Cardiac and renal dysfunction in rats with CRS2 are ameliorated after DMB treatment; 3) DMB treatment improves myocyte hypertrophy as well as cardiac and renal fibrosis in rats with CRS2; 4) DMB treatment reduces expression of proinflammatory cytokines in the heart and kidney in rats with CRS2. Taken together, these observations suggest that elevated circulating TMAO levels contribute to cardiac and renal dysfunction in rats with CRS2 and that attenuation of circulating TMAO levels can ameliorate cardiac and renal injury and prevent the progression of CRS2.

Growing evidence from clinical and experimental studies reveals that TMAO is a key contributor to the pathogenesis of both chronic HF and CKD (Mamic et al., 2020). TMAO is formed *via* a two-step process. First, gut microbes enzymatically produce trimethylamine (TMA) from the TMA-containing dietary constituents such as choline, phosphatidylcholine or carnitine; TMA is then absorbed into the blood and converted to TMAO in the liver by flavin-containing monooxygenase (FMO) enzymes, primarily FMO3 (Anselmi et al., 2020). Under normal physiologic conditions, the kidneys rapidly clear circulating TMAO via urinary excretion (Velasquez et al., 2016). Alterations in gut microbiota (dysbiosis) or impaired renal function can promote TMAO synthesis or reduce TMAO clearance from kidney, leading to elevated circulating TMAO levels (Kitai et al., 2016; Xu et al., 2017). Previous studies have shown that patients with systolic HF have elevated circulating TMAO levels, which are identified as a prognostic biomarker and associated with increased long-term mortality independent of traditional biomarkers of risk in the HF population (Tang et al., 2014; Albert and Tang, 2018). TMAO or its precursor choline treatment of mice with pressure-overload–induced HF causes more severe pulmonary edema, cardiac enlargement, myocardial fibrosis and reduction in LV ejection fraction (Organ et al., 2016). Inhibition of TMAO production has been shown to improve myocardial infarction-induced HF in rats (Li X. et al., 2019). Moreover, a prospective cohort study of 521 stable subjects with CKD showed marked increases in circulating TMAO levels, which were associated with a 2.8-fold elevated mortality risk (Tang et al., 2015). Experimental study reported that TMAO or its precursor choline treatment of mice induced renal tubulointerstitial fibrosis and dysfunction (Mafune et al., 2016). Our previous study showed that circulating TMAO levels were increased in a mouse model of diet-induced obesity and that inhibition of TMAO alleviated renal interstitial fibrosis and obesity-associated CKD (Sun et al., 2017). However, few studies to date have examined the role of TMAO in the progression of CRS2. An early study in patients with stable chronic systolic HF reported that circulating TMAO levels were positively correlated with B-type natriuretic peptide levels and negatively correlated with the estimated glomerular filtration rate (eGFR) (Tang et al., 2014). A new study showed that circulating TMAO levels were significantly increased in HF patients with preserved ejection fraction (HFpEF) compared with controls (Guo et al., 2020). In addition, circulating TMAO levels were inversely correlated with eGFR and HFpEF patients with impaired renal function (eGFR <60 ml/min/1.73 m^2^) had greater levels of TMAO than those with normal eGFR (≥60 ml/min/1.73 m^2^) (Guo et al., 2020). In the present study, we found that MI + STNx rats, compared with SHAM + SHAM controls, had significant higher levels of serum TMAO at week 6, which provides the direct evidence that circulating TMAO levels are elevated in CRS2. Patients with HF have been shown to have alterations in the composition of gut microbiota (Kuehn, 2019), and perhaps this might promote TMAO generation. Renal injury might cause reductions in renal excretion of TMAO, leading to increased circulating TMAO (Stubbs et al., 2016). A previous study reported that circulating TMAO levels were around 26 μM in HF rats 8 weeks after coronary artery ligation-induced MI (Li X. et al., 2019). In our study, the circulating TMAO levels were 37 ± 4 μM in CRS2 rat model 6 weeks after coronary artery ligation-induced MI and 2 weeks after STNx, which were obviously higher than those levels in HF rats 8 weeks after coronary artery ligation-induced MI. These data suggest that increased TMAO levels in CRS2 rat model are due to both MI-induced HF and STNx-induced renal injury. Of note, circulating TMAO levels in VEH-treated MI + STNx rats were increased further at week 14 compared with week 6, suggesting the accumulation of TMAO in the circulation over time. In agreement with previous study (Savira et al., 2020b), our results showed that MI + STNx rats exhibited impaired cardiac function and remodeling as indicated by reduced LVEF and LVFS and increased LVESV and LVEDV, and renal dysfunction as evidenced by increased levels of serum creatinine and urinary KIM-1 and decreased creatinine clearance at week 6. The further decreases in LVFS, creatinine clearance and the further increases in LVESV, LVEDV, levels of serum creatinine and urinary KIM-1 and elevated proteinuria were observed in VEH-treated MI + STNx rats at week 14 compared with those at week 6. These changes suggest the cardiac and renal dysfunction was exacerbated over time, which was consistent with the alterations in circulating TMAO levels. Importantly, we found that treatment with TMAO inhibitor DMB attenuated circulating TMAO levels, prevented the exacerbations in LVFS, LVESV, LVEDV, levels of serum creatinine, urinary KIM-1 creatinine clearance and proteinuria in MI + STNx rats at week 14. In addition, DMB treatment of MI + STNx rats also improved LV and RV hypertrophy and pulmonary congestion (indexes of MI-induced HF) as evidenced by decreases in LV/tibial length, RV/tibial length and lung/tibial length, respectively. These results suggest that elevated circulating TMAO levels contribute to the progression of CRS2 and that inhibition of TMAO production can ameliorate the CRS2.

Increased cardiac fibrosis is associated with augmented myocardial stiffness, cardiomyocyte necrosis, arrhythmias, sudden cardiac death and unfavorable prognosis, and plays an important role in the remodeling process that leads to HF (Espeland et al., 2018). Increased renal fibrosis is a key contributor to the progressive decline in renal function with time (Hewitson, 2009; Qiu et al., 2019). Consistent with previous studies (Liu et al., 2013; Savira et al., 2020b), VEH-treated MI + STNx rats had significant increases in cardiac and renal interstitial fibrosis and myocyte hypertrophy, which were associated with upregulation in gene expression of key pro-hypertrophic and pro-fibrotic markers. DMB treatment of MI + STNx rats significantly reduced cardiac and renal interstitial fibrosis and myocyte hypertrophy, which were accompanied by decreases in gene expression of pro-hypertrophic and pro-fibrotic markers. These results indicate that inhibition of TMAO production prevent the progression of CRS2 by reducing cardiac and renal fibrosis. Indeed, previous studies from our laboratory and others have shown that DMB treatment attenuates cardiac and renal fibrosis and improves cardiac and renal dysfunction in high-fat diet-induced obese animals (Chen et al., 2017; Sun et al., 2017).

Inflammation has been suggested to account for cardiac and renal fibrosis (Hewitson, 2009; Chen et al., 2017; Sun et al., 2017; Savira et al., 2020b). To further determine the mechanism by which DMB treatment attenuates cardiac and renal fibrosis in the rat model of CRS2, we measured gene expression of proinflammatory cytokines at week 14. We found that gene expression of IL-8 in the heart and gene expression of IL-6 and IL-8 in the kidney were significantly increased in VEH-treated MI + STNx rats but were reduced in DMB-treated MI + STNx rats. These results suggest that inhibition of TMAO production reduces cardiac and renal fibrosis in the rat model of CRS2 by decreasing inflammatory responses.

The nuclear factor (NF)-kB and Smad3 signaling pathways are strongly implicated in the process of cardiac and renal interstitial fibrosis and myocyte hypertrophy (Gaspar-Pereira et al., 2012; Savira et al., 2017; Pan et al., 2020). Activation of NF-kB can induce expression of proinflammatory cytokines to initiate inflammatory responses, resulting in cardiac and renal interstitial fibrosis and myocyte hypertrophy (Savira et al., 2020b; Pan et al., 2020). Activation of Smad3 by TGF-β1 leads to nuclear translocation of Smad3 to activate hypertrophic and pro-fibrotic genes, causing cardiac and renal interstitial fibrosis and myocyte hypertrophy (Pan et al., 2020). TMAO has been reported to promote cardiac remodeling and renal interstitial fibrosis by activating NF-kB and Smad3 (Tang et al., 2015; Yu et al., 2018; Li Z. et al., 2019; Zhang et al., 2021), whereas inhibition of TMAO reduces activation of NF-kB and Smad3, preventing cardiac remodeling and renal interstitial fibrosis in multiple cardiac and renal diseases (Li Z. et al., 2019; Zhang et al., 2021). Thus, inhibition of TMAO prevents the progression of cardiac and renal dysfunction in CRS2 rats probably by suppressing activation of NF-kB and Smad3 signaling pathways.

Several limitations of the study should be acknowledged. First, the number of animals in each group was small, which might have limited the ability to reach significance in some analyses given the biological variability that could occur. Second, circulating TMAO levels were not measured in CRS2 rats prior to STNx procedure, we therefore could not provide direct evidence that increases in circulating TMAO levels in CRS2 rats were due to both surgical interventions. Finally, the pathological mechanism of TMAO action on CRS2 was not determined, although we speculate that TMAO inhibitor might exert the beneficial effects on CRS2 by attenuating activation of NF-kB and Smad3 signaling pathways.

In conclusion, the present study demonstrates that rats with CRS2 have elevated circulating TMAO levels, which are associated with the exacerbation of cardiac and renal dysfunction. Attenuation of circulating TMAO levels alleviates cardiac and renal injury and prevents the progression of CRS2 by suppressing inflammation. The findings suggest that circulating TMAO may be a novel target for therapeutic intervention in the CRS2.

## Data Availability

The original contributions presented in the study are included in the article/Supplementary Material, further inquiries can be directed to the corresponding author.
